# Are children who do not receive their first Measles Mumps and Rubella (MMR1) vaccination by 24 months more likely to share a household with older non-vaccinated children? Linked household-level analysis of primary care electronic health records (EHRs).

**DOI:** 10.23889/ijpds.v7i3.2059

**Published:** 2022-08-25

**Authors:** Milena Marszalek, Nicola Firman, Marta Wilk, Kate Homer, Gill Harper, Carol Dezateux

**Affiliations:** 1 Queen Mary University of London

## Objectives

Only 89.4% of UK children receive MMR1 by age 24 months. We investigated whether children not receiving MMR1 by this age were more likely to share a household with children also not vaccinated by this age, and its variation by sex, ethnic background and area-level deprivation.

## Approach

We identified 208,907 children born between 2013-2019 and eligible for MMR1 from the primary care EHRs of 1,192,630 children registered to a general practitioner in North East London between 2001-2021.  We estimated the proportion of children and 95% Confidence Interval (CI) receiving MMR1 between 12 and 24 months of age. We calculated prevalence ratios (PR) by sex, ethnicity, and deprivation (Index of Multiple Deprivation (IMD) quintiles).  We identified all children with the same pseudonymised Unique Property Reference Numbers (pUPRNs) at the MMR1 date. We are completing analyses to calculate mutually adjusted PRs and examine household-level associations in timeliness of MMR1.

## Results

Overall, 172,319 (82.5%) of 208,907 eligible children received an MMR1 between age 12-24 months. Non-receipt of MMR1 by 24 months was less likely in children from South Asian ethnic backgrounds (PR 0.74; 95% CI: 0.68,0.79), and more likely in those from Black ethnic backgrounds (1.43;1.31,1.56) and in those living in the most deprived IMD quintile (1.83;1.49,2.23). We identified 137,919 children with the same pUPRNs at the MMR1 date, comprising 69,892 boys (50.7%) and 41,846 (30.3%), 32,631 (23.7%), 10,793 (7.8%), 12,246 (8.9%) and 40,403 (29.3) from White, South Asian, Black, Mixed/Other and Missing ethnic backgrounds respectively. We are calculating adjusted PRs and will estimate associations between non-receipt of MMR1 in the youngest and oldest children within a household.

## Conclusion

Our findings suggest that non-receipt of MMR1 by 24 months is ethnically patterned and more likely in areas of higher deprivation. Household-level analyses provide actionable insights into the characteristics of measles-susceptible households and opportunities for data-enabled primary care interventions to reduce vaccination inequalities and prevent measles outbreaks.

